# The Unseen Poor

**Published:** 1885-01

**Authors:** 


### THE UNSEEN POOR.

It is forgotten that while to the lowest scale of human life poverty is a hard lot somewhat tempered by habit, to higher grades of society poverty is really a crime. They dare not show to their neighbors and acquantances any outward evidences of their poverty—they dare not reveal the terrible punchings and struggles they go through to keep their little home together, or the anxiety they suffer in raising the little rent they have to pay weekly for a humble lodging in a respectable house and neighborhood. The poor gentleman, the poor lady, the poor clerk out of employment, must maintain their respectability, for their pecuniary ruin means also social ruin. There is a point in certain grades of human existence where respectability becomes a burden and a tax. It is all very well to say "There is menial labor open to them." There is no greater cant abroad than the affectation that menial labor is a disgrace. But menial labor requires skill, and unless a man or woman be reared to it he or she is valuless in that capacity. To be a competent navvy or laborer requires a certain muscular development and training. To be a skilled carpenter or bricklayer requires as much knowledge, skill, and nicety of touch as many callings of higher repute. I am purposely putting aside all considerations of the natural and actual horror and pain felt by all refined natures at contact with sordid surroundings and coarsely vulgar associates. But to the well-bred and educated man or woman all this means trial and suffering quite unknown to the inhabitants of a slum. The deserving poor, the poor who get no sympathy, do not all live in slums. The popular journalist can make no sensational articles on the lives of men who conceal their sufferings under decent black coats and nearly starve in dingy two-pair backs. The suffering is silent, it is not advertised. In the privacy of their poorly furnished rooms, the tears may be bitter, the sighs heavy, but the world knows nothing of all that. The poor tradesman, ruined, perhaps, by no fault of his own—crushed by competing with huge capitalists--who will set him on his legs again? A careful study of the annual statistics of suicides will show that nearly all the "cases" found are respectably dressed. The inhabitants of slums seldom commit suicide. The most powerful incentives to suicide are shame, anxiety, and mental suffering.—*All the Year Round.*

